# The American Association for the Advancement of Science

**Published:** 1869-10

**Authors:** 


### EDITORIAL DEPARTMENT.

---

*The American Association for the Advancement of Science*—This Association met at Salem, Mass, in August last, and continued one week, being largely attended by scientific gentlemen from all sections of the country. Professor Agassiz, as usual, was very entertaining and was the life of the meeting.

More than one hundred papers were read, many of which were listened to with the deepest interest. The "Antiquity of Man" was the title of one of the papers read, which, although it shed no new light upon the subject, was yet a very interesting one. Prof. Marsh exhibited some fossil bones which were found at the bottom of a well, and had been forwarded to the Association under the label of "Human Bones found in Tertiary Deposits." Prof. Marsh, however, proved them to belong to an extinct species of horses, whose normal height was but two feet. One entire evening was devoted to some strange experiments with the human heart, conducted by Drs. Groux and Upham. "Dr Groux was born without any breast bone, and the action of the heart was shown in him as in no other human being. He exposed his breast, and by sundry ingenious arrangements, including a connection with a telegraphic instrument, "revealed to ear and eye the threefold motion and sound of the heart." He also, says a report of the evening's examination, showed how its position changed by strong inhalation and exhalation, and finally carried out the dangerous experiment of causing its motion entirely to cease for some seconds, as was tested by two physicians, one holding the pulse and the other listening with a stethoscope on the breast. Then the telegraphic apparatus was put in connection with a hospital twenty miles away in Boston, and the heart-beats of the physicians and patients there made to sound out sharply on a bell, and to reveal themselves also to the eye by means of the flashings of a calcium light on the wall. We counted' says Dr. Patton, the sixty heart strokes a minute of a healthy man, and then the one hundred and thirty strokes of a fever patient, and then the irregular, interrupted pulsations of one with heart disease. There was something solemnly impressive in these experiments so close to the seat of life."

